# Patterns of adverse childhood experiences and associations with prenatal substance use and poor infant outcomes in a multi-country cohort of mothers: a latent class analysis

**DOI:** 10.1186/s12884-022-04839-0

**Published:** 2022-06-22

**Authors:** Chad Lance Hemady, Lydia Gabriela Speyer, Aja Louise Murray, Ruth Harriet Brown, Franziska Meinck, Deborah Fry, Huyen Do, Siham Sikander, Bernadette Madrid, Asvini Fernando, Susan Walker, Michael Dunne, Sarah Foley, Claire Hughes, Joseph Osafo, Adriana Baban, Diana Taut, Catherine L. Ward, Vo Van Thang, Pasco Fearon, Mark Tomlinson, Sara Valdebenito, Manuel Eisner

**Affiliations:** 1 Social Work Department, University of Edinburgh, Edinburgh, UK; 2 School of Social and Political Science, 15a George Square, Edinburgh, EH8 9LD UK; 3 Department of Psychology, University of Edinburgh, Edinburgh, UK; 4 Faculty of Humanities, North-West University, Potchefstroom, South Africa; 5 Moray House School of Education and Sport, University of Edinburgh, Edinburgh, UK; 6 Faculty of Health, Queensland University of Technology, Brisbane, Australia; 7 Department of Primary Care & Mental Health, University of Liverpool, Liverpool, UK; 8 Global Institute of Human Development, Shifa Tameer-E-Millat University, Islamabad, Pakistan; 9 Child Protection Unit, University of the Philippines, Manila, Philippines; 10 Department of Paediatrics, Faculty of Medicine, University of Kelaniya, Colombo, Sri Lanka; 11 Caribbean Institute for Health Research, The University of the West Indies, Kingston, Jamaica; 12 Australian Centre for Health Law Research, Queensland University of Technology, Brisbane, Australia; 13 Centre for Family Research, University of Cambridge, Cambridge, UK; 14 Department of Psychology, University of Ghana, Accra, Ghana; 15 Department of Psychology, Babes-Bolyai University, Cluj-Napoca, Romania; 16 Department of Psychology, University of Cape Town, Cape Town, South Africa; 17 Institute for Community Health Research, Hue University, Hue, Vietnam; 18 Division of Psychology & Language Sciences, University College London, London, UK; 19 Department of Global Health, Institute of Life Course Health Research, Stellenbosch University, Cape Town, South Africa; 20 School of Nursing and Midwifery, Queens University, Belfast, UK; 21 Institute of Criminology, University of Cambridge, Cambridge, UK; 22 Jacobs Center for Productive Youth Development, University of Zurich, Zurich, Switzerland

**Keywords:** Adverse childhood experiences, Latent class analysis, Maternal health, Neonatal health, Prenatal substance use, Intergenerational transmission of adversity

## Abstract

**Background:**

This paper enumerates and characterizes latent classes of adverse childhood experiences and investigates how they relate to prenatal substance use (i.e., smoking, alcohol, and other drugs) and poor infant outcomes (i.e., infant prematurity and low birthweight) across eight low- and middle-income countries (LMICs).

**Methods:**

A total of 1189 mother-infant dyads from the Evidence for Better Lives Study cohort were recruited. Latent class analysis using the Bolck, Croon, and Hagenaars (BCH) 3-step method with auxiliary multilevel logistic regressions was performed.

**Results:**

Three high-risk classes and one low-risk class emerged: (1) *highly maltreated* (7%, *n* = 89), (2*) emotionally and physically abused with intra-familial violence exposure* (13%, *n* = 152), (3), *emotionally abused* (40%, *n* = 474), and (4) *low household dysfunction and abuse* (40%, *n* = 474). Pairwise comparisons between classes indicate higher probabilities of prenatal drug use in the *highly* maltreated and *emotionally abused* classes compared with the *low household dysfunction and abuse* class. Additionally, the *emotionally and physically abused with intra-familial violence exposure* class had higher probability of low birthweight than the three remaining classes.

**Conclusion:**

Our results highlight the multifaceted nature of ACEs and underline the potential importance of exposure to childhood adversities on behaviors and outcomes in the perinatal period. This can inform the design of antenatal support to better address these challenges.

**Supplementary Information:**

The online version contains supplementary material available at 10.1186/s12884-022-04839-0.

## Background

Felitti and colleagues provided seminal evidence for a strong gradient relationship between adverse childhood experiences (ACEs) and poor health [1]. Research in this field has burgeoned [2] to document the various pathways that link ACEs, detrimental health outcomes throughout the individual’s life-course [3, 4], and the subsequent generations [5]. In recent years, research has focused on illuminating the mechanisms of intergenerational transmission of adversity to inform intervention targets and tackle vicious cycles of disadvantage.

One important pathway may be via the impact of maternal behaviors during pregnancy. Embryonic and fetal exposure to teratogens during this critical period can disrupt growth and development which may lead to long term deficits [6]. There is evidence for an association between maternal ACEs and prenatal smoking and other drug use [7], alcohol use [8], and offspring low birthweight and infant prematurity [9]. For instance, Smith and colleagues found an inverse relationship between maternal ACEs and infant birthweight and gestation age, with prenatal smoking identified as a mediating pathway [7]. However, causal effects should not be overestimated as the relationship between prenatal smoking and offspring outcomes are often muddied by other factors, such as timing, extent and duration of exposure, and confounding environmental / genetic factors[10–12].

While most investigations of links between ACEs and substance use adopt a cumulative risk approach that assumes an additive and linear step-wise relationship between risk factors and outcomes examined [13], some studies suggest that exposure to one to three ACEs have similar levels of effects compared to no ACEs [15]. Likewise, while effects of individual risks are often assumed to be equal in magnitude [15], recent studies suggest that certain ACE items (e.g., parental divorce) have become less predictive of poor outcomes. Indeed, cumulative ACE scores, by themselves, do not sufficiently express the heterogeneity of high-risk profiles and the potential synergistic and interactive effects between risk factors [15]. To this point, a review by Briggs and colleagues illustrated that certain combinations of ACEs interact to increase overall risk, undercutting the rationale for using a sum score in policy and practice settings [16]. By contrast, person-centered analyses, such as latent class analyses (LCA) that are free from the unwarranted assumptions of the cumulative risk approach, have the potential to provide a multidimensional characterization of the entanglement of adversities and their links to key outcomes.

The value of LCA to meet this need has been increasingly demonstrated in ACEs studies that use the technique to parse the heterogeneity in ACE exposure into potentially meaningful risk profiles [13]. LCA is a finite mixture model that is used to define latent subgroups within a population through a set of manifest or observed variables [17]. For instance, one study explored clusters of latent ACEs and their associations with internalizing disorders among US school-aged children and identified five classes: *Income hardship* (10.6%), *Parental divorce or separation* (23.2%), *Mental illness or substance abuse exposure* (12.6%)*, High ACEs* (8.8%), and *No ACEs* (44.8%) [14]. Additionally, their findings suggested that the *Mental illness or substance abuse exposure* class and *High ACEs* were more likely to report any childhood internalizing disorder compared to the *No ACEs* class.

### The current study

This study examined the number and characterization of latent classes of childhood adversity in a prospective birth cohort study involving mother-infant dyads residing across eight diverse low- and middle-income countries (LMICs). Additionally, we explored the relationships between latent ACEs, prenatal substance use, and poor infant outcomes (i.e., infant prematurity and low birthweight). We hypothesized that high-risk latent ACE classes, characteristic of high levels of child maltreatment and household dysfunction, are more likely to be associated with prenatal substance use and adverse infant outcomes. Additionally, we hypothesized that salient risk factors (e.g., sexual abuse, physical abuse) have synergistic effects and are more predictive of adverse outcomes than other ACE domains (e.g., parental divorce).

To the best of our knowledge, this is the first study to explore how the latent typologies of maternal ACEs link to prenatal substance use and poor infant outcomes in LMICs. Of the.

few studies to apply LCA to determine typologies of risk of early childhood adversity using the nine domains of the pioneering ACE study [1], most involve high-income countries (HICs). This study therefore adds to the literature through its focus on the impact of childhood adversity in families residing in LMICs, often exposed to further adversity, such as economic deprivation, and under-resourced / less well-established health and social care systems.

## Method

### Participants

All participants were taking part in the Evidence for Better Lives Study (EBLS) [18], an ongoing multi-country prospective birth cohort study comprising 1208 mother-infant dyads that aims to examine the environmental, societal, interpersonal, and biological mechanisms that impact child development. A key priority of EBLS was to establish a multicentric study focused on LMICs. Eight country sites across the Latin-American and Caribbean region (Kingston, Jamaica), Europe (Cluj-Napoca, Romania), Africa (Koforidua, Ghana and Worchester, South Africa), the Indian Subcontinent (Tarlai Kalan, Pakistan and Ragama, Sri Lanka) and Asia (Hue, Vietnam and Valenzuela, Philippines) participated in the birth cohort. Participants were recruited from local health centers during antenatal check-ups, using the following inclusion criteria for mothers: i) third trimester of pregnancy; ii) above 18 years of age; iii) residing within the defined geographical locations. Measurements were carried out between 29–40 weeks of gestation (Wave 1), with a follow-up when the offspring was between two to six months (Wave 2). Informed consent was collected from all participants. A total of 1529 expectant mothers were approached during the Wave 1 of this study with 1208 consenting to participate, giving a participation rate of 79% (Evidence for Better Lives Consortium, 2019). After removing duplicates, outliers, and participants with twin neonates, the final sample was *N* = 1189. The Evidence for Better Lives Study protocol for recruitment and collection of data was approved by the University of Cambridge, School of Social Sciences Ethics Board (18/180) as well as all relevant ethics boards in the data collection sites. The study was carried out in accordance with the Declaration of Helsinki regarding the ethical conduct of medical research involving human subjects. See https://www.vrc.crim.cam.ac.uk/vrcresearch/EBLS [19] for more detail about the data collection process.

### Measures

#### Adverse Childhood Experiences – International Questionnaire (ACE-IQ)

The 29-item ACE-IQ [20] assesses experiences of childhood adversity during the participants’ first 18 years of life. The current study adapted the ACE-IQ into an abridged 19-item version grouped into nine abuse (e.g., sexual abuse) or household dysfunction (e.g., parental incarceration) domains. This study used the binary version of the ACE score analysis where the 19 items were grouped into the nine domains and coded dichotomously (Once, A Few Times, Many Times = 1, Never = 0) [21]. Cronbach’s alpha for these scores was 0.81. A summary of the different instruments used, specific set of questions, and respective response qualifiers are included in the Additional File (Table S1).

#### Alcohol, Smoking and Substance Involvement Screening Test (ASSIST)

The *Alcohol, Smoking and Substance Involvement Screening Test* (ASSIST) [22] was used to measure the health outcome of interest: prenatal substance use, analyzed by examining the prevalence of substance use during the past six months. Tobacco and alcohol use were coded dichotomously and scored as 1 if the participants have *ever* used tobacco and alcohol during pregnancy, respectively. Tobacco and alcohol use was further categorized with heavy tobacco use and heavy alcohol use coded as 1 if the participant claimed to have used either ‘monthly’, ‘weekly’ or ‘daily or almost daily’ during the past six months. Since there is a relatively small number who used other drugs (i.e., cannabis [*n* = 16], cocaine [*n* = 31], amphetamine [*n* = 7], inhalants [*n* = 12], sedatives or sleeping pills [*n* = 5], hallucinogens [*n* = 3], and opioids [*n* = 3]) during pregnancy for non-medical use, each drug was coded as 1 if they claimed to use at least once during the past six months. A sum score for the eight substances was created and coded dichotomously (0 = no other drug use during pregnancy, 1 =  ≥ 1 other drug use during pregnancy).

#### Maternal follow-up measures

Between three to six months postpartum, participants reported on offspring birth weight (‘how big was your child when he/she was born?’) and maternal week of birth (‘how many weeks pregnant were you when your baby was born?’). Following the definition of the World Health Organization (WHO) on infant prematurity [23], births before 37 completed weeks of pregnancy were categorized as 1 while those more than 37 weeks were categorized as 0. Moreover, WHO [23] defined ‘low birthweight’ as birthweight less than 2.5 kg (irrespective of gestational age at birth); this variable was coded dichotomously with 1 =  < 2.500 and 0 =  ≥ 2.500 kg. We used these measures as they provide the clearest clinical interpretability.

#### Covariates

The following covariates were adjusted for in the analyses: age, perceived socioeconomic status (SES), and educational level. Perceived SES was assessed using the *MacArthur Scale of Subjective Social Status* [24], which captures SES indicators using a 10-point ‘social ladder’. The participants placed an ‘X’ on the rung of their perceived ranking in the social hierarchy, with the score ranging from 0 – 10 (lowest to highest in social hierarchy). Highest level of education attainment was adapted from the *Demographic and Health Survey* (DHS) using a 10-points scale ranging from lowest (‘none at all’) to highest (‘University Doctoral Degree’). Maternal SES and educational level were coded as an ordinal variable with values ranging from 1–10. The country of residence (*N* = 8) was treated as a second-level variable to account for the nested design of the study.

### Missing data

Missing data were handled using multiple imputations (MI) using the mice package in *R* [25]. The package generates multiple imputations through chained equations where each missing value is imputed using a distinct model. A two-level regression model with the country of residence as the second-level variable was used with 20 imputations. Continuous variables were imputed using imputation by a two-level normal model; binary variables by imputation by a two-level logistic model; and ordinal data by imputation of most likely value within class.

### Statistical procedure

Latent Class Analysis using the Bolck, Croon, and Hagenaars (BCH) 3-step method with auxiliary multilevel logistic regressions on the distal outcomes were performed using *MPlus 8.5* [26]. This approach required two separate runs: the first run estimated the latent class unconditional model with the BCH weights, covariates, and distal outcomes saved on an auxiliary dataset. The second run estimated the multilevel logistic regression model conditional on the latent class variable saved as BCH weights [27]. Models were estimated using robust maximum likelihood estimation. For the first step it was necessary to select a model with an optimal number of classes. An increasing number of classes were included and this was terminated when the criteria suggesting an empirically under-identified model was satisfied, including: i) maximum likelihood values could no longer be replicated; ii) class overlap and over-extraction is evident; ii) failure of estimation algorithm to converge a large proportion of random sets of starting values [17]. The fitted models were then compared to select an optimal model.

There is not a singular method used when determining the ‘optimal’ model; thus, a host of fit indices were utilized: Bayesian Information Criterion (BIC), Sample-size Adjusted Bayesian Information Criterion (SABIC), Consistent Akaike Information Criterion (CAIC) and Approximate Weight of Evidence Criterion (AWE) [28]. The model with the lowest value was judged to have the relatively superior fit. In assessing the relative fit of the model, that is, comparing two models’ representation of the data, the $\rho$*-values* of the Lo-Mendel-Rubin likelihood ratio test (LMR-LRT) and bootstrapped likelihood ratio test (BLRT) were examined. and the Bayes Factor (BF). A Bayes Factor (BF) of less than 3 suggest weak support for the model with fewer classes (K -1), while a BF more than 3 but less than 5 suggests moderate support and a BF with more than 10 suggest strong evidence [28]. Substantive and pragmatic criteria also guided the model selection, including assessing the stability of the models, considering the relative sizes of the classes per model, and applying the principle of parsimony when comparing two classes with marginal differences in fit indices [28].

Characterization of the resultant classes of the selected model was based on posterior membership probabilities and strong item-class relationships. Strong item-class relationships must fulfil two features: within-class homogeneity and between-class separation [20]. High class homogeneity, or the similarity of people in their respective classes to endorse or not endorse an item, is indicated by a high (> 0.7) and low (< 0.3) model estimated probabilities of item endorsement, respectively [38]. On the other hand, class separation, or how different individuals across latent classes in their item responses, can be determined through the odds ratio of item probabilities; a large *OR* (> 5) or small *OR* (< 0.2) indicate a high degree of separation between classes *j* and *k.* The item should be distinct across at least one pair of classes among the *K* latent classes [20].

To examine the associations of the latent classes and the covariates on the distal outcomes, the second run of the BCH method was employed by estimating the multilevel logistic regression models conditional on the latent class variable saved as BCH weights [23]. The BCH method allows for pairwise significance tests of threshold differences using the Wald test while holding class membership constant. Pairwise comparisons were interpreted as statistically significant at *p* < 0.05.

## Results

### Participant demographics

The study was composed of 1189 women in their third trimester of pregnancy (mean age 28.7; standard deviatio*n* = 5.79). Almost half (48%) completed secondary school or high school, and more than half (56%) scored 4–6 on the *MacArthur Subjective Social Status Scale*. More than a third (39%) had reported exposure to ≥ 4 ACEs while 11%, 15%, and 6%, reported tobacco, alcohol, and other drug use during the past six months, respectively. Finally, 7% reported that their infants were born prematurely while 8% reported that their infants had low birthweight.. Demographic characteristics (Table S2), frequencies and relative frequencies of each ACE item endorsement (Table S3) and the cluster membership per country (Table S4) are outlined in the Additional File.

### ACE risk profiles

**Fig. 1:**
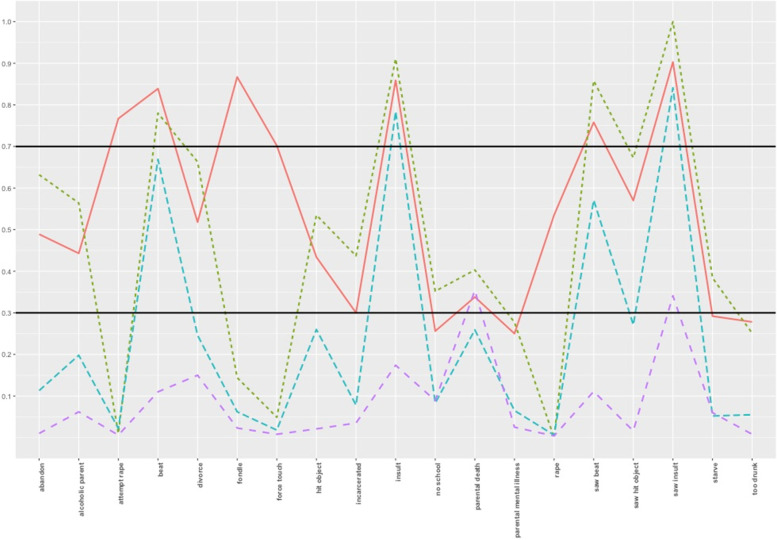
Maternal adverse childhood experiences profile plot. Class 1: *highly maltreated children* (red line); Class 2: *emotionally and physically abused with familial violence exposure* (dashed line, blue); Class 3: *emotionally abused* (two-dashed line, purple); Class 4: *low household dysfunction and abuse* (dotted line; green)

The class enumeration process was terminated at the *K* = *6* class model which exhibited empirical under-identification. Table 1 summarizes the fit indices. The 4-class model had the lowest BIC value (18,399.132) but the 5-class model had the lowest SABIC (18,113.173), CAIC (18,129.743), and AWE (18,129.743) values. BF values of the 4-class model (> 4) suggest moderate support over the 5-class model, while the adjusted (LMR-LRT), signifies that the 4-class model was optimal. Finally, in contrast to the 4-class model, the 5-class model did not seem to yield a particularly distinct class in terms of patterns of endorsement. Thus, judging from the evaluative diagnostic above and following the principle of parsimony, the 4-class model was chosen as the final unconditional model. Interpretation of the four classes were primarily dependent on the model-estimated, class-specific item response probabilities, class homogeneity and class separation. In Table 2, the item response probabilities are bolded suggesting high homogeneity within class (> 0.7 or < 0.3). In Table 3, item response odds ratios are bolded suggesting high degrees of class separation in relation to the two latent class comparisons (> 5 or < 0.2). Figure 1 shows the profile plot of the four latent ACEs subgroups.

**Table 1:** Fit statistics and classification coefficients

K	par	*LL*	BIC	SABIC	CAIC	AWE	BF_k,k+1_	LMR-LRT *p-value*	BLRT *p-value*
1	19	-10,401.019	20,936.687	20,876.336	20,879.51	20,889.01	< 3		-
2	39	-9296.006	18,868.394	18,744.515	18,751.04	18,770.54	< 3	** < .001**	< .001
3	59	-9049.660	18,517.437	18,330.031	18,339.90	18,369.40	< 0	< .05	< .001
4	79	-8919.640	**18,399.132**	18,148.198	18,161.42	18,200.92	> 4	< .001	< .001
5	99	-8863.024	18,427.635	**18,113.173**	18,129.74	18,179.24	** > 7**	0.60	< .001
6	119	-8812.199	18,467.719	18,089.729	**18,109.64**	**18,169.14**	-	0.74	< .001

Note: *K* Number of classes, *par* parameters, *LL* Log-likelihood, *BIC* Bayesian Information Criterion, *SABIC* Sample-size adjusted BIC, *CAIC* Consistent Akaike Information Criterion, *AWE* Average Weight of Evidence Criterion, *BF*_*k,k*+*1*_ Bayes Factor, *LMR-LRT* Lo-Mendell-Rubin Likelihood Ratio Test, *BLRT* Bootstrap Likelihood Ratio Test

**Bold** indicates best fit for each respective test statistic

**Table 2:** Model-estimated, Class-specific Item Response Probabilities Based on the Unconditional 4-class LCA

Adverse Childhood Experiences	Class 1 *n* = 89 (7%)	Class 2 *n* = 152 (13%)	Class 3 *n* = 474 (40%)	Class 4 *n* = 474 (40%)
**Did you live with a household member who:**				
1. was a problem drinker or alcoholic, or misused street or prescription drugs?	0.446	0.556	**0.201**	**0.059**
2. was depressed, mentally ill or suicidal?	**0.250**	**0.275**	**0.066**	**0.022**
3. was ever sent to jail or prison?	**0.302**	0.434	**0.079**	**0.035**
4. Were your parents ever separated or divorced?	0.511	0.666	**0.243**	**0.150**
5. Did your mother, father or guardian die?	0.341	0.389	**0.263**	0.354
**Did you see or hear a parent or a household member in your home:**				
6. being yelled at, screamed at, sworn at, insulted or humiliated?	**0.901**	**1.00**	**0.840**	**0.336**
7. being slapped, kicked, punched or beaten up?	**0.756**	**0.861**	0.573	**0.108**
8. being hit or cut with an object, such as a stick (or cane), bottle, club, knife, whip etc.?	0.567	0.671	**0.270**	**0.016**
**Did a parent, guardian or other household member:**				
9. yell, scream or swear at you, insult or humiliate you?	**0.857**	**0.926**	**0.777**	**0.174**
10. threaten to, or actually, abandon you or throw you out of the house?	0.480	0.649	**0.109**	**0.010**
11. spank, slap, kick, punch or beat you up?	**0.836**	**0.787**	0.664	**0.110**
12. hit or cut you with an object, such as a stick (or cane), bottle, club, knife, whip etc.?	0.428	0.539	**0.257**	**0.021**
**Did someone:**				
13. touch or fondle you in a sexual way when you did not want them to?	**0.875**	**0.147**	**0.062**	**0.022**
14. make you touch their body in a sexual way when you did not want them to?	**0.711**	**0.050**	**0.018**	**0.008**
15. attempt oral, anal, or vaginal intercourse with you when you did not want them to?	**0.776**	**0.012**	**0.022**	**0.006**
16. actually have oral, anal, or vaginal intercourse with you when you did not want them to?	**0.534**	**0.011**	**0.004**	**0.005**
17. How often did your parents/guardians not give you enough food even when they could easily have done so?	**0.293**	0.376	**0.053**	**0.060**
18. How often were your parents/guardians too drunk or intoxicated by drugs to take care of you?	**0.279**	**0.243**	**0.056**	**0.007**
19. How often did our parents/guardians not send you to school even when it was available?	**0.259**	0.339	**0.086**	**0.090**

Note: **Bold** indicates high class homogeneity

**Table 3:** Model-Estimated item response odds ratios for all latent class comparisons bason on the 4-class unconditional LCA

ACE	Class 1 vs 2	Class 1 vs 3	Class 1 vs 4	Class 2 vs 3	Class 2 vs 4	Class 3 vs 4
**Did you live with a household member who:**						
1. was a problem drinker or alcoholic, or misused street or prescription drugs?	0.64	3.20	**12.76**	**5.00**	**19.84**	3.98
2. was depressed, mentally ill or suicidal?	0.88	4.68	**14.83**	**5.32**	**16.83**	3.16
3. was ever sent to jail or prison?	0.56	**5.04**	**11.88**	**8.95**	**21.13**	2.35
4. Were your parents ever separated or divorced?	0.52	3.25	**5.93**	**6.21**	**11.34**	1.83
5. Did your mother, father or guardian die?	0.81	1.45	0.95	1.77	1.16	0.65
**Did you see or hear a parent or a household member in your home:**						
6. being yelled at, screamed at, sworn at, insulted or humiliated?	**0.00**	1.73	**17.92**	**52,095.76**	***	**10.38**
7. being slapped, kicked, punched or beaten up?	0.50	2.31	**25.69**	4.63	**51.60**	**11.12**
8. being hit or cut with an object, such as a stick (or cane), bottle, club, knife, whip etc.?	0.64	3.56	**82.96**	**5.51**	**128.89**	**23.38**
**Did a parent, guardian or other household member:**						
9. yell, scream or swear at you, insult or humiliate you?	0.48	1.71	**28.35**	3.57	**59.21**	**16.57**
10. threaten to, or actually, abandon you or throw you out of the house?	0.50	**7.58**	**88.99**	**15.15**	**177.95**	**11.74**
11. spank, slap, kick, punch or beat you up?	1.38	2.58	**41.20**	1.86	**29.91**	**16.03**
12. hit or cut you with an object, such as a stick (or cane), bottle, club, knife, whip etc.?	0.64	2.17	**34.13**	3.38	**53.28**	**15.76**
**Did someone:**						
13. touch or fondle you in a sexual way when you did not want them to?	**40.94**	**107.04**	**306.11**	2.61	**7.48**	2.86
14. make you touch their body in a sexual way when you did not want them to?	**46.97**	**135.19**	**309.11**	2.87	**6.58**	2.29
15. attempt oral, anal, or vaginal intercourse with you when you did not want them to?	**274.82**	**157.21**	**574.31**	0.57	2.09	3.65
16. actually have oral, anal, or vaginal intercourse with you when you did not want them to?	**101.42**	**311.79**	**252.05**	3.07	2.49	0.81
17. How often did your parents/guardians not give you enough food even when they could easily have done so?	0.69	**7.40**	**6.47**	**10.74**	**9.40**	0.88
18. How often were your parents/guardians too drunk or intoxicated by drugs to take care of you?	1.20	**6.49**	**51.10**	**5.40**	**42.51**	**7.88**
19. How often did our parents/guardians not send you to school even when it was available.?	0.69	3.70	3.53	**5.44**	**5.20**	0.96

Note: **Bold** indicates high between-class separation

#### Class 1 – highly maltreated children

Class 1, with an estimated proportion of 7% *(n* = *89)*, had high homogeneity in 13 out of 19 items. It is characterized by a high likelihood of endorsing experiences of witnessing intra-familial violence, experiencing physical, emotional, and sexual abuse. Since rape (item 16) occurs so infrequently in this sample, an item endorsement probability of 0.53 is considered a defining characteristic for this class. In terms of experiencing sexual violence, Class 1 is well separated from the three remaining classes. Class 1 is well separated from Class 4 in all items except item 5 (parental death) and item 19 (was not allowed to attend school).

#### Class 2 – emotionally and physically abused children with intra-familial violence exposure

Class 2 with an estimated proportion of 13% *(n* = *152)*, had high homogeneity in 10 items. Individuals in this class had a high probability of endorsing witnessing a family member being beaten (0.861), being yelled at, screamed at, sworn at, insulted or humiliated (1.00). This class also had a high-class membership of experiencing verbal abuse (0.926) and physical abuse (0.787) from a family member. Alternatively, Class 2 had a low probability of endorsing items related to parental mental illness and items related to sexual violence. Class 2 is well separated from Class 4 in all but three items (parental death [item 5]; attempted rape [item 15], rape [item 16]). Class 2 is also well separated from Class 3 in 11 out of 19 items.

#### Class 3 – emotionally abused children

Class 3, with an estimated proportion of 40% *(n* = *474)*, exhibits high homogeneity for class membership in all except two items (witnessing a parent being beaten [0.573]; experiencing being beaten [0.664]). This class is characterized by low probabilities of endorsement for almost all items except for two: item 6) witnessing a parent or household member in their home being yelled at, screamed at, sworn at, insulted or humiliated (0.840); and item 9) being yelled at, screamed at, sworn at, insulted or humiliated by a parent or a family member (0.777). For these two items, Class 3 is well separated from Class 4. Class 3 is well separated from Class 2 in item 6 but not item 9. Finally, Class 3 is not well separated from Class 1 in both items.

#### Class 4 – low household dysfunction or abuse

Class 4, with an estimated proportion of 40% *(n* = *474)*, had high homogeneity in all but one item (parental death = 0.354). Across all items, class membership has low probabilities of endorsement suggesting that this class has low levels of household dysfunction and/or abuse.

#### Latent classes and distal outcomes

The class-specific threshold values and significance tests for each distal outcome are presented in Table 4. It is worthy to note that high threshold values are associated with low probabilities [26]. χ^2^tests revealed that, adjusting for maternal SES, education level, and age, the *highly maltreated* class and the *emotionally abused* class had significantly greater probability of prenatal other drug use compared to the *low household dysfunction and abuse* class [26]. Additionally, the class with *intra-familial violence exposure* class had significantly higher probability of experiencing neonatal low birthweight compared to the three remaining classes; yet overall, the logit threshold values of all classes suggest low probability. No other pairwise comparisons were significant for the remaining outcomes.

**Table 4:** Significant differences between class-specific thresholds of distal outcomes using χ^2^tests

Class (threshold)	Class 1 (*n* = 89)	Class 2 (*n* = 152)	Class 3 (*n* = 474)
Prenatal alcohol use			
c1 (1.086)			
c2 (0.864)	0.018		
c3 (1.645)	0.035	0.017	
c4 (2.651)	0.008	-0.010	-0.027
Prenatal heavy alcohol use			
c1 (2.372)			
c2 (2.581)	-0.401		
c3 (3.216)	-0.061	-0.019	
c4 (4.279)	-0.094	-0.053	-0.033
Prenatal tobacco use			
c1 (1.119)			
c2 (1.137)	0.015		
c3 (2.144)	0.036	0.020	
c4 (2.791)	0.003	-0.012	-0.033
Prenatal heavy tobacco use			
c1(1.463)			
c2(1.472)	-0.007		
c3(2.919)	-0.053	-1.552	
c4(3.347)	-0.006	0.002	0.553
Prenatal other drug use			
c1(2.010)			
c2(2.501)	-0.058		
c3(2.744)	0.064	0.122	
c4(3.701)	-0.131*	-0.071	**-0.196†**
Low birth weight			
c1(2.221)			
c2(2.031)	-0.143*		
c3(2.833)	0.014	**0.163 ‡**	
c4(2.094)	-0.087	0.044	-0.1017*
Infant prematurity			
c1(2.150)			
c2(2.446)	0.069		
c3(2.414)	0.035	-0.034	
c4(2.437)	0.027	-0.042	-0.008

The values under column one are class specific threshold values for each distal outcome. The values in columns two to four are the results from the pairwise significance tests

^*^ = *P* < 0.05, † = *P* < 0.01, ‡ = *P* < 0.001

## Discussion

To the best of our knowledge, this is the first adverse childhood experiences (ACEs) study to focus on a cohort of mothers residing in eight diverse LMICs to provide a more global perspective on the impact of ACEs. Using the Evidence for Better Lives Study (EBLS) dataset, the number and characterizations of latent ACEs and their associations with prenatal substance use and poor infant outcomes were explored. The findings suggested high prevalence and co-occurrence of maternal ACEs in this cohort, with 39% having experienced ≥ 4 ACEs which is higher than in ACE studies involving pregnant women in HICs [29].

A model with four distinct latent classes was judged optimal for these data, with classes labelled: *highly maltreated* (7%), the *emotionally and physically abused with intra-familial violence exposure* (13%), the *emotionally abused* (40%), and the *low household dysfunction and abuse* (40%). Overall, classes had high within-class homogeneity and between-class heterogeneity.

Previous studies have used the cumulative risk approach to demonstrate a dose–response relationship between maternal ACEs and prenatal smoking and alcohol use [30, 31]. By contrast, in this EBLS cohort, there was insufficient evidence for an association between membership in the *highly maltreated* class and the outcomes of interest, indicating that structural factors such as cultural norms may play a protective role. For instance, normative gender roles, religious practices, and social taboos may preclude women from smoking, consuming alcoholic beverages, and using other substances for recreational use [32–34].

Prenatal other drug use was present in 6% of the study sample; this is comparable to other ACE studies involving a cohort of pregnant women residing in a HIC (3.1%) [35]. Multilevel logistic regressions suggested that, more broadly, there is low probability of prenatal illicit drug use across the different classes; these findings are inconsistent with previous research that suggest a dose–response relationship between maternal ACEs and prenatal other drug use [29]. However, the pairwise differences between classes suggest that the *highly maltreated class* and the *emotionally abused* class, but not the *intra-familial violence exposed* class, had greater probability of prenatal illicit drug use compared to the normative class. Indeed, child sexual abuse, physical abuse, and exposure to intra-familial violence are synergistically reactive forms of ACEs [16]; this may partially explain why the *highly maltreated* class had increased risk compared to the normative class. However, what is less clear is why the three high-risk ACE classes were statistically indistinguishable from each other. Similarly, and inconsistent with previous studies [7, 9], there is insufficient evidence to suggest that the high-risk classes were associated with either infant prematurity or low birth weight. Yet, pairwise differences between classes suggest that the *intra-familial violence exposed* class had significantly higher probability of experiencing neonatal low birth weight compared to the three remaining classes. This is incongruous with both the additive and linear assumption of the cumulative risk approach and the synergistic effects model given that the *highly maltreated* class had the same forms of risks as the *intra-familial violence exposed* class but with a higher number of ACEs. This highlights the potential importance of various parameters of risk exposure (severity, duration, and chronicity) [36].

A review of developmental resilience science literature shows that some individuals are able to positively adapt in the face of cumulative and severe exposure to childhood adversities [37]. Indeed, there is growing evidence that resilience during the perinatal period can moderate the impact of ACEs on maternal functioning [38, 39]. In fact, a recent study involving the EBLS cohort of mothers found that ACEs had a positive relationship with fetal attachment [40], which could suggest that exposure to ACEs may have a ‘buffering’ effect on the mothers’ compassion and empathy towards their unborn child. It may be the case that despite being exposed to a high number of synergistically reactive ACEs, these mothers-to-be are able to disrupt the cycle of adversity by fostering post-traumatic growth and resilience. It is important to note that resilience is not circumscribed within the biological and psychological systems of individuals but extends to the myriad interactions of macro-systems including culture, society, and ecology [37]. Social support during pregnancy is an example of a protective factor that can moderate the risk of poor birth outcomes [41, 42]. Given that ACEs can be moderated by other factors which may accentuate or attenuate detrimental outcomes, further investigation on the health inequities and social determinants, including the wide range of community-level and structural-level factors is warranted [15].

The study has some potentially important implications for policy and practice. Primarily, our results highlight the importance of considering the multifaceted nature of ACEs. Concurrent with the continuous growth of the ACE field of study is the increasing number of proponents for universal ACEs screening as part of standard medical assessment [43] and routine prenatal care [44]. However, Robert Anda, one of the researchers of the first ACE study, cautions against using the cumulative ACE score as a diagnostic tool in clinical decision making to predict health risks of individuals and to determine the need for treatment and services [45]. One of the biggest costs to screening is overtreatment and service referral for people who have already recovered or may not truly benefit from them [46]. This will be burdensome for health system foundations, particularly in LMICs where inadequate resources and ineffective organizational referral systems remain as persistent challenges [47]. Our study adds to these concerns that screening using a cumulative risk score is unlikely to best capture women’s experiences of ACEs and associated risk. Rather, profiles of ACE exposure (not just overall levels) may be important to capture to estimate women’s risk of adverse perinatal behaviors and outcomes.

Despite the concern raised about their use as screens, ACE questionnaires or similar tools can be used to introduce the sensitive topic to expectant mothers, followed by a systematic clinical assessment of the nature of their childhood adversity with detailed discussion of, among other things, developmental chronicity, frequency, severity, and how this exposure is currently impacting their behavior and wellbeing [16, 45]. Our results suggest that, dependent on the outcome of these assessments, follow-up assessment for substance use may be merited in contexts where this is not already routine.

This study has several limitations. First, convenience sampling constrained generalizability to the wider population. Second, principal investigators from each site selected health care providers that provided antenatal check-ups. Only mothers who were able to visit the local health centers had the chance to participate in the study; mothers who were not able to attend routine check-ups and in turn might be more vulnerable and at higher risk of poor pregnancy outcomes were not recruited. Third, the psychometric measures were translated from English into nine local languages: Afrikaans, IsiXhosa, Romanian, Tagalog, Tamil, Twi, Romanian, Sinhala, Urdu, and Vietnamese. Thus, it is possible that the translated items may not have been homogenous in their meaning across the nine languages. To give an example, the ACE item on parental death was coded as missing for Pakistan because the translation of the item in Urdu was not representative of the original question. This may partially explain why class homogeneity has been low for this item across all classes. Fourth, information on exposure to ACEs were collected from retrospective and self-administered reports, which have been shown to exhibit high false-negative scores [48]. However, this is a widely accepted data collection method and currently, there are no alternative methods available (e.g., administrative records); even if there were, they would probably underreport even more significantly. Respondents may also have had difficulties recollecting certain childhood occurrences or may have opted not to disclose traumatic experiences [49]. In some instances, the participants were chaperoned by a family member. It is possible that the women were reluctant to share sensitive information even though their accompanying family member was a distance away during the interview. Fifth, we did not have a sufficient sample size to compare findings across our sites. Future studies making explicit cross-country comparisons would be valuable to illuminate potential contextual influences on ACE patterns and links to outcomes in the perinatal period. Finally, the use of various substances were aggregated into one variable (‘other drugs’) due to their low prevalence of use; yet, it should be noted that different drugs can have different impacts on birth outcomes [50] and future research should examine these separately.

## Conclusion

The results further our understanding of the dynamic and multifaceted nature of ACEs. Contrary to previous research, there were insufficient evidence linking exposure to multiple ACEs to prenatal substance use and poor infant outcomes. The findings highlight the importance of bring more attention to various parameters of risk exposure (i.e., severity, duration, chronicity). Additionally, more ACE research grounded on LMICs with focus on exploring the impact of socioecological factors can help optimize interventions for both mother and child.

## Supplementary Information

**Additional file 1:** **Table S1.** Instruments used, questions, and response categories. **Table S2.** Demographic characteristics of the sample from the Evidence for Better Lives Study (n = 1 189). **Table S3.** Adverse childhood experiences item endorsement frequencies and relative frequencies. **Table S4.** Class counts and proportions for the latent classes based on their most likely latent class membership.

## Data Availability

The datasets used and/or analysed during the current study are available from the corresponding author on reasonable request.

## Abbreviations

ACEs: Adverse Childhood Experiences
ACE-IQ: Adverse Childhood Experiences – International Questionnaire
AIC: Akaike Information Criterion
ASSIST: Alcohol, Smoking and Substance Involvement Screening Test
AWE: Approximate Weight of Evidence Criterion
BCH: Bolck, Croon, and Hagenaars
BF: Bayes Factor
BIC: Bayesian Information Criterion
BLRT: Bootstrapped likelihood ratio test
CAIC: Consistent Akaike Information Criterion
DHS: Demographic and Health Survey
EBLS: Evidence for Better Lives Study
HICs: High income countries
LCA: Latent Class Analysis
LL: Log-likelihood
LMICs: Low- and middle-income countries
LMR-LRT: Lo-Mendel-Rubin likelihood ratio test
LTB: Lanza, Tan, and Bray approach
MI: Multiple Imputations
ML: Maximum Likelihood three-step approach
SABIC: Sample-size Adjusted Bayesian Information Criterion
SES: Socioeconomic Status
WHO: World Health Organization
